# *In situ* embedding dual-Fe nanoparticles in synchronously generated carbon for the synergistic integration of magnetic resonance imaging and drug delivery†

**DOI:** 10.1039/d0na00714e

**Published:** 2020-09-26

**Authors:** Hui Zhang, Jianping Zhang, Qianqian Zhang, Xiaofeng Liu, Yongtai Yang, Yun Ling, Yaming Zhou

**Affiliations:** Shanghai Key Laboratory of Molecular Catalysis and Innovative Materials, Department of Chemistry, Fudan University Shanghai 200433 China ymzhou@fudan.edu.cn; Department of Nuclear Medicine, Fudan University Shanghai Cancer Center Shanghai 200032 China; Zhuhai Fudan Innovation Institute Zhuhai Guangdong 519000 China

## Abstract

*In situ* incorporating versatile magnetic iron nanoparticles into ordered mesoporous carbon (OMC) by means of synthetic methodology for functional integration is a great challenge. Inspired by the phenomenon of uniovular twins in nature, a homometallic [Fe_9_(μ_3_-O)_4_(O_3_PPh)_3_(O_2_CCMe_3_)_13_] ({Fe_9_P_3_}) cluster was synthesized and used as the ovulum to *in situ* produce dual-Fe nanoparticle (γ-Fe_2_O_3_ and Fe(PO_3_)_3_)-functionalized OMC (dual-Fe/OMC). *In vitro* magnetic resonance imaging (MRI) studies showed a longitudinal relaxation (*r*_1_) and transverse relaxation (*r*_2_) of 9.74 and 26.59 mM^−1^ s^−1^ with a *r*_2_/*r*_1_ ratio of 2.73 at 0.5 T. The MRI performances were further examined by mouse model with a subcutaneous HeLa tumor. In addition, the low cytotoxicity, considerable loading capacity and delivery of doxorubicin hydrochloride (DOX) were also studied *in vitro*. These results demonstrate the feasibility of the concept of uniovular twins in the one-pot preparation of dual-Fe/OMC for functional integration.

## Abstract 2

Inspired by the phenomenon of uniovular twins in nature, dual-Fe nanoparticles functionalized ordered mesoporous carbon have been one-pot facilely prepared, achieving the synergistic intergration of MRI effects with drug delivery property.

## Graphical abstract

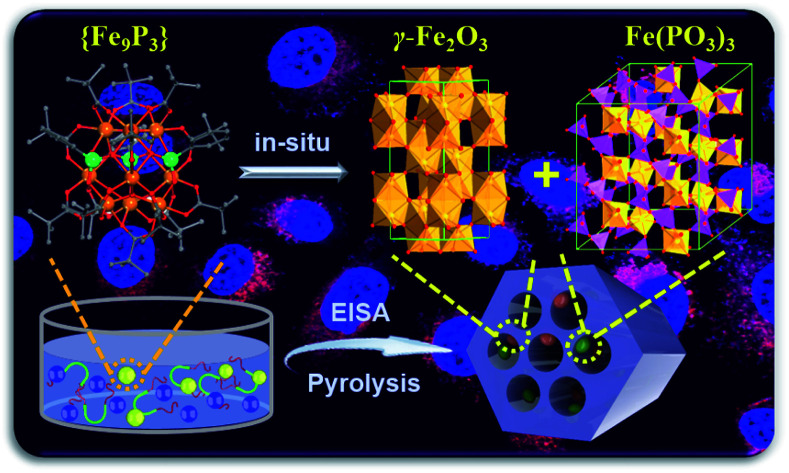

## Introduction

Integrating magnetic nanoparticles into porous materials is a feasible way to develop theranostic nanomaterials for the combination of magnetic resonance imaging (MRI) and drug delivery systems (DDSs).^1–5^ Generally, iron (Fe) or gadolinium (Gd) compounds that possess superparamagnetic or paramagnetic properties are clinical candidates for MRI.^6–9^ As for DDSs, ordered mesoporous carbons (OMCs) are regarded as a kind of promising candidates owing to their large pore volumes, high surface areas and excellent biocompatibility.^10–14^ Thus, structures in the form of a magnetic core coated by OMCs have been proposed for functional integration.^15–17^ However, only one single small magnetic core is not sufficient to produce substantial MRI contrast effect. Although increasing the size of the magnetic core is practical for the improvement of the MRI contrast effect, the drug-carrying capacity and biocompatibility are compromised in this way. In addition, it would lead to an unexpected transition from superparamagnetic to ferromagnetic for iron oxide when the size is larger than 20 nm.^18,19^ An optional structure-model is to uniformly embed abundant magnetic nanoparticles into OMCs.

The co-assembly of magnetic precursors with carbon sources is a facile way to achieve such form of composites. Gd- (for *T*_1_-weighted MRI) and Fe-based (for *T*_2_-weighted MRI) nanoparticles have each been incorporated into OMCs.^20–22^ Consequently, Gd- and Fe-based nanoparticle co-functionalized OMCs have been explored to further achieve the combined *T*_1_–*T*_2_ MRI with DDSs.^23–25^ However, Gd-based compounds would produce a risk of severe nephrogenic systemic fibrosis.^26,27^ Biocompatible Fe/OMCs are therefore more desired in the exploration of combined biofunctions. Besides downsizing the iron oxide nanoparticles to enhance the *T*_1_ signal (at the expense of the *T*_2_ effect),^28–31^ introducing another kind of Fe-based paramagnetic nanoparticle is a way that is worth considering. However, producing dual-Fe nanoparticles synchronously with the generation of OMC in the one-pot pyrolysis reaction remains a significant challenge by means of the synthetic methodology.

In this work, inspired by the phenomenon of uniovular twins in nature, a homometallic [Fe_9_(μ_3_-O)_4_(O_3_PPh)_3_(O_2_CCMe_3_)_13_] ({Fe_9_P_3_}) cluster was synthesized and used as the ovulum for a proof-of-principle study. The co-assembly of {Fe_9_P_3_} with resol was achieved *via* the evaporation-induced self-assembly (EISA) method. This was followed by the one-pot carbonization process, leading to the *in situ* generation of dual-Fe (γ-Fe_2_O_3_ and Fe(PO_3_)_3_) nanoparticles functionalized OMC (denoted as dual-Fe/OMC). The dual-Fe nanoparticles had a size of around 5 nm, and were well dispersed and embedded in the OMC matrix. *In vitro* MRI studies demonstrate a longitudinal relaxation (*r*_1_) of 9.74 mM^−1^ s^−1^ and a transverse relaxation (*r*_1_) of 26.59 mM^−1^ s^−1^ with a *r*_2_/*r*_1_ ratio of 2.73 at 0.5 T. Based on the mouse model with a subcutaneous tumor, the MRI tomography contrast effects were also examined. In addition, the *in vitro* studies further demonstrate its low cytotoxicity, considerable loading capacity and capability of delivering doxorubicin hydrochloride (DOX).

## Experimental section

### Materials and characterization

Poly(ethylene oxide)-*block*-poly(propylene oxide)-*block*-poly-(ethylene oxide) triblock copolymer (Pluronic F127, *M*_w_ = 12 600, PEO_106_PPO_70_PEO_106_) were purchased from Acros Corp. Fe(NO_3_)_3_·9H_2_O, pivalic acid, phenylphosphonic acid (C_6_H_5_PO_3_H_2_), acetonitrile (MeCN), trimethylamine, phenol, formalin solution (37 wt%), sodium hydroxide, hydrochloric acid, tetrahydrofuran (THF), ethanol (EtOH), ether (Et_2_O) and doxorubicin hydrochloride (DOX) were purchased from Aladdin. Dulbecco minimum essential medium (DMEM) and 4′,6-diamidino-2-phenylindole (DAPI) were purchased from Sigma-Aldrich. All chemicals were used as received without any further purification.

Thermal Gravimetric Analysis (TGA) was carried out on a Mettler Toledo TGA/SDTA 851 thermoanalyzer from 40 to 600 °C at a heating rate of 10 °C min^−1^ under N_2_/airflow. Powder X-ray diffraction (PXRD) data were recorded on a Bruker D8 Advance diffractometer at 40 kV, 40 mA with Cu K_α_ radiation (*λ* = 1.5406 Å). Small-angle X-ray scattering (SAXS) measurements were taken on a Nanostar U SAXS system (Bruker, Germany) using Cu K_α_ radiation (40 kV, 35 mA). Transmission Electron Microscopy (TEM) measurements were conducted on a JEM-2100 microscope (JEOL, Japan) operated at 200 kV. N_2_ sorption was measured on an ASAP 2020 gas adsorption apparatus (Micromeritics) at 77 K. X-ray photoelectron spectroscopy (XPS) was recorded on a Perkin Elmer PHI 5000C ESCA system (Perkin Elmer, USA). Inductively coupled plasma atomic emission spectroscopy (ICP-AES) were measured using a PerkinElmer Avio 200 Optical Emission Spectrometer. Before gas absorption, the samples were degassed under vacuum at 200 °C for 10 h. The UV-vis absorbance spectra were collected on a Perkin Elmer UV Spectrometer Lambda 750S. The confocal laser-scanning microscopy (CLSM) measurement was performed with an Olympus FluoView FV1000 confocal laser scanning microscope and a 60× (oil-immersion) objective lens. Magnetic measurements were carried out on a MPMS (SQUID) VSM magnetometer equipped with a 7 T magnet. Zero Field Cooled (ZFC) and field cooled (FC) experiments were carried out by measuring the static magnetization at a field of 100 Oe as the temperature was swept from 10 to 350 K at a rate of 2 K min^−1^ in a sample cooled in the absence of an applied field (ZFC), and in a sample cooled under an applied field (FC). The magnetization isotherm was collected at 300 K between −2 and 2 T. The *in vitro* MRI scans were performed on a 0.5 T MR system (Shanghai Niumai MesoMR23-060H-I), a 1.5 T MR system (Siemens Aera 1.5 T MRI Scanner, Erlangen, Germany) and a 3 T MR system (Siemens Prisma 3 T MRI Scanner, Erlangen, Germany).

### Sample preparation

#### {Fe_9_P_3_} cluster

A mixture of [Fe^III^_3_(μ_3_-O)(O_2_C^*t*^Bu)_6_(HO_2_C^*t*^Bu)_3_](O_2_C^*t*^Bu)^35^ (0.06 g, 0.05 mmol), C_6_H_5_PO_3_H_2_ (0.008 g, 0.05 mmol) and acetonitrile (4 mL) was placed in a 10 mL teflon-line autoclave, and was stirred at room temperature for 5 min. The mixture was then heated at 150 °C for 24 h, followed by cooling down to room temperature. Red crystals [Fe_9_(μ_3_-O)_4_(O_3_PPh)_3_(O_2_CCMe_3_)_13_] ({Fe_9_P_3_}) were collected by filtration. Yield: 60% (based on Fe).

#### Dual-Fe/OMC

The products were prepared *via* EISA method, and then followed by one-pot pyrolysis. Typically, 0.1 g of Pluronic F127 was dissolved in 5 g of THF. Then, 0.6 g of the resol precursor solution (20 wt% in THF) was added. After stirring for 10 min at room temperature, 5 g of THF dissolved with *n* mg of {Fe_9_P_3_} was dropped into the above mixture. After stirring for another 2 h, the mixture was cast onto a Petri dish (diameter = 150 mm), followed by evaporation of THF for 8 h at room temperature. Then, they were subjected to thermocuring at 100 °C for 24 h. Finally, the as-made products were scraped off the dishes and carbonized at a defined temperature *T* for 3 h with a heating rate of 1 °C min^−1^ under N_2_ flow. The obtained composites were denoted as dual-Fe/OMC-*n-T*. Accordingly, the dosage *n* was varied from 6 to 30 mg with an increment of 6 mg in the case of *T* = 600 °C. At *n* = 24 mg, the carbonization temperature *T* was varied from 600 to 800 °C with an increment of 100 °C.

#### γ-Fe_2_O_3_/OMC

The preparation is similar to that of dual-Fe/OMC-24-600, except {Fe_9_P_3_} is replaced with iron acetylacetonate. It should be mentioned that the dosage of iron acetylacetonate was calculated based on the molar amount of γ-Fe_2_O_3_ in the dual-Fe/OMC-24-600.

#### Fe(PO_3_)_3_/OMC

The preparation is similar to that of dual-Fe/OMC-24-600, except {Fe_9_P_3_} is replaced with iron phenylphosphonate. It should be mentioned that the dosage of iron phenylphosphonate was calculated based on the molar amount of Fe(PO_3_)_3_ in the dual-Fe/OMC-24-600.

All of the above-prepared samples were surface-activated with 15% H_2_O_2_ solution for 2 h at room temperature on a shaker.

### MR imaging studies

#### In vitro

The composites were dispersed in deionized water at various iron concentrations. *T*_1_-weighted MR images were acquired using a multi-slice, multi-echo sequence under the following parameters: TR/TE = 200/14 ms, 256 × 192 matrices, 100 × 100 mm^2^ fields of view, Sweep Width (SW) = 20 kHz, and a slice thickness of 3 mm. *T*_2_-weighted MR images were acquired using a fast spin-echo sequence to reduce the acquisition time under the following parameters: TR/TE = 3000/100 ms, 256 × 192 matrices, 100 × 100 mm^2^ fields of view, SW = 20 kHz, a slice thickness of 3 mm. The specific relaxivity values of *r*_1_ and *r*_2_ were calculated through the curve fitting of 1/*T*_1_ (s^−1^) and 1/*T*_2_ (s^−1^) *vs.* iron concentration (mmol L^−1^).

#### In vivo

Female BALB/c nude mice were purchased from the Shanghai Slac Laboratory Animal Co., Ltd. Animal experiments were carried out according to the protocols approved by the Animal Care and Use Committee of Fudan University. Mice bearing HeLa tumors were prepared by subcutaneous injection of 2 × 10^6^ HeLa cells into the right back of the hind leg of the female BALB/c nude mouse. A series of sequential MRI at varying time points (pre 0 h, 0.25 h, 1 h, 2 h and 4 h) was acquired on MRI equipment (7.0 T Bruker, small animal) after intratumoral injection of DOX@dual-Fe/OMC-24-600 (10 mg Fe per kg).

### *In vitro* cell assay

#### Cell cytotoxicity

The cytotoxicity of dual-Fe/OMC-24-600 on HeLa cells was evaluated by Cell Counting Kit-8 (CCK-8) assay. HeLa cells were harvested by trypsinization and seeded into a 96-well cell culture plate at 1 × 10^4^ per well, and incubated for 24 h at 37 °C under 5% CO_2_. Then, the HeLa cells were co-cultured with 1–160 μg mL^−1^ of dual-Fe/OMC-24-600 for 4 h. The CCK-8 kit was then (10 μL per well) added to each well, and incubated at 37 °C for 1.5 h. Enzyme dehydrogenase in the living cells was oxidized with this kit to the orange carapace. The quality was assessed calorimetrically by using a multi-reader (TECAN, Infinite M200, Germany). The measurements were based on the absorbance values at 450 nm. The viability of the cell was then calculated by the following eqn (1):1
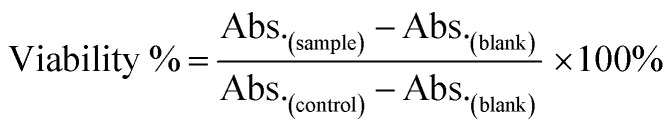


#### Drug loading

The 30 mg of dual-Fe/OMC-24-600 was dispersed into 2 mL (1 mg mL^−1^) of DOX solution. The mixture was stirred for about 12 h at room temperature under dark condition. Then, the DOX-loaded sample was collected by centrifugation and washed with PBS solution. The supernatant and washed solutions were collected. The concentration of DOX in the collection solution was determined at its maximum absorbance of 481 nm by UV/VIS spectrophotometer. These steps were repeated for the DOX-loaded dual-Fe/OMC-24-600 until the DOX concentration of the solution was steady. The drug loading capacity (LC) was calculated by the following eqn (2):2
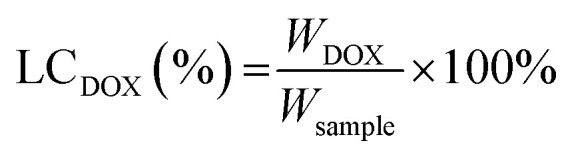
where *W*_DOX_ was the loaded weight of DOX, and *W*_sample_ was the weight of the activated dual-Fe/OMC-24-600 sample.

#### Cellular uptake evaluation

HeLa cells (5 × 10^4^) were seeded on 10 mm confocal glass bottom dishes for 24 h. Then, the cell culture medium was changed to fresh DMEM with DOX@dual-Fe/OMC-24-600 (100 μg mL^−1^). After incubation at 37 °C for 0.5 h, the cells were rinsed three times with PBS, followed by fixing with paraformaldehyde solution for 15 min at 4 °C. Then, the nuclei of the HeLa cells were stained by DAPI (1 μg mL^−1^). Finally, the cells were washed with PBS solution twice. The stained cells were imaged using an Olympus FluoView FV1000 confocal microscope (ex/em: 488/580–680 nm for DOX, ex/em: 405/550–650 nm for DAPI). Following the same procedure, the HeLa cells were treated with DOX@dual-Fe/OMC-24-600 for different time periods (1 h, 2 h, and 4 h).

## Results and discussion

### Preparation and characterization of dual-Fe/OMC

**Fig. 1:**
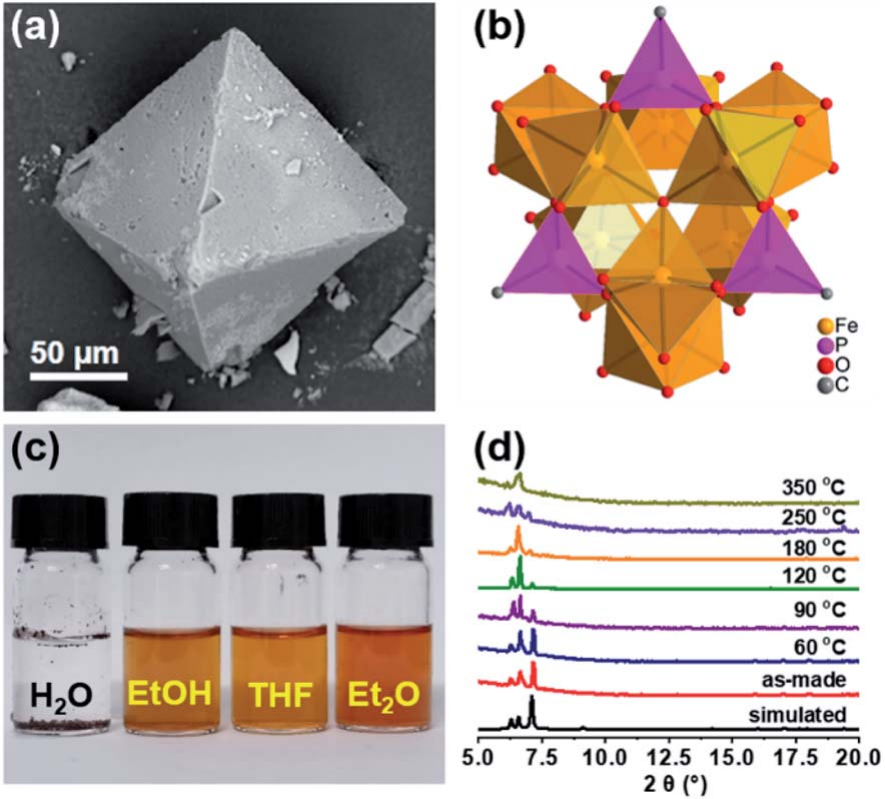
(a) An SEM image of crystalline {Fe_9_P_3_} in an octahedral geometry. (b) The crystal structure of {Fe_9_P_3_} from CCDC showing Fe and P in a polyhedron geometry, while other organic groups are omitted for clarity. (c) Optical images of {Fe_9_P_3_} in different solvents. (d) PXRD patterns of as-made {Fe_9_P_3_}, as well as its temperature-dependent crystal structure transformation.

In order to follow the method of evaporation-induced self-assembly (EISA) to prepare the functional ordered mesoporous carbon (OMC),^32^ metallic precursors bearing properties of good solubility in organic solvents and synchronous decomposition with resol are required. A homometallic cluster of {Fe_9_P_3_} consisting of phenylphosphonate and pivalate ligands was therefore screened out from the CCDC database, and synthesized according to literature reports.^33–35^ Scanning Electron Microscopy (SEM), powder X-ray diffraction (PXRD), and Fourier transform infrared spectroscopy (FT-IR) characterizations demonstrated the successful isolation of the {Fe_9_P_3_} cluster (Fig. 1 and S1†). Its solubility was then carefully examined by ultrasonic dispersion of {Fe_9_P_3_} (10 mg) in 3 mL of the individual solvents, H_2_O, EtOH, THF and Et_2_O, revealing its solubility property in common organic solvents. Thermal gravimetric analysis (Fig. S1c†) and variable-temperature PXRD characterizations showed that there was no obvious weight loss or crystal phase transformation as the temperature increased from 30 to 350 °C, indicating an excellent physical property that is capable of avoiding uncontrollable migration before pyrolysis. Furthermore, the PXRD pattern of the residues obtained at 600 °C revealed the presence of diffraction peaks assigned to γ-Fe_2_O_3_ (JCPDS card no. 39-1346) and Fe(PO_3_)_3_ (JCPDS card no. 38-0109) (Fig. S1d†). All of these results suggest that the {Fe_9_P_3_} cluster fulfills the requirements of the EISA method in the preparation of dual-Fe/OMC under the concept of uniovular twins.

**Scheme 1:**
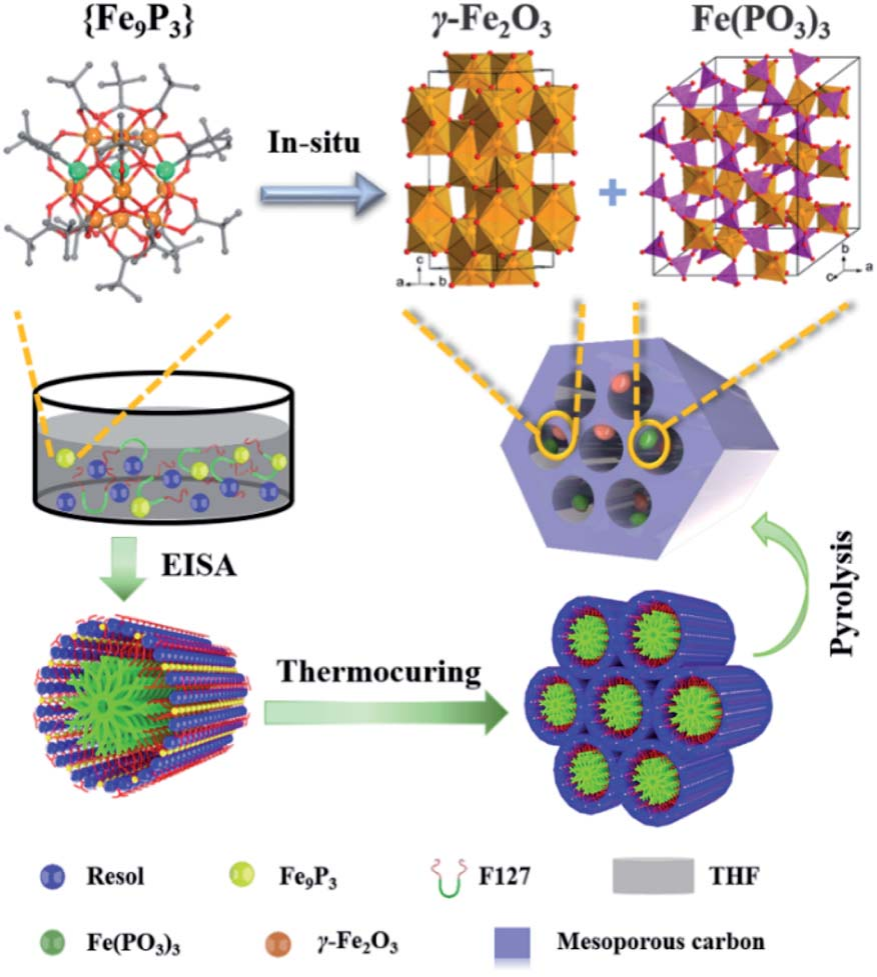
Schematic illustration of the preparation procedure for dual-Fe/OMC.

**Fig. 2:**
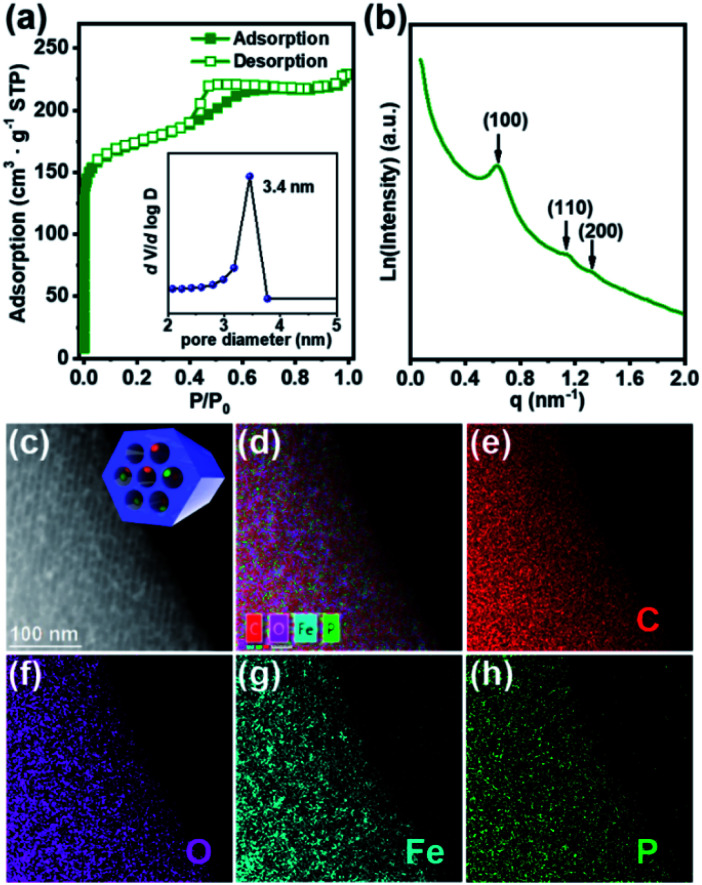
(a) N_2_ isotherm of dual-Fe/OMC-24-600 at 77 K (inset: pore size distribution by BJH). (b) SAXS spectrum of dual-Fe/OMC-24-600. (c) HAADF-STEM image. (d) Overlay maps of C, O, P and Fe elements. The corresponding EDS maps of C (e), O (f), Fe (g), and P (h).

Brief procedures for the preparation of dual-Fe/OMC is portrayed in Scheme 1. A reddish-brown film was obtained by EISA of resol, F127 and {Fe_9_P_3_}. Pyrolysis of the film at a defined temperature *T* resulted in the isolation of a black powder carbon composite, which was named as dual-Fe/OMC-*n-T* accordingly (*n*: doping amount of {Fe_9_P_3_} in mg, *T*: pyrolysis temperature). As a representative example, characterizations of dual-Fe/OMC-24-600 are described here. Its porosity was evaluated by N_2_ sorption at 77 K (Fig. 2a), which showed a type-IV isotherm with a H_2_-type hysteresis loop. The Brunner–Emmet–Teller (BET) surface area was calculated to be 662 m^2^ g^−1^ with a mean pore size of 3.4 nm by BJH method based on the desorption isotherm. The small-angle X-ray scattering (SAXS) pattern revealed three reflection peaks at 0.6 nm^−1^, 1.1 nm^−1^, and 1.3 nm^−1^ assigned to the (100), (110) and (200) planes of the 2D hexagonal *P*6*mm* structure, respectively (Fig. 2b). Together with the results of the transmission electron microscopy (TEM) images (Fig. S2†) and Raman spectroscopy (Fig. S3†), the formation of dual-Fe/OMC can be concluded. The presence of homogenously dispersed Fe and P in the OMC was identified by elemental mapping (Fig. 2c). The elements were further revealed to be in the state of Fe^3+^ and P^5+^ by XPS (Fig. S4†) with a ratio close to 3 : 1 by ICP-AES (Table S1†).^36–38^ This is consistent with the stoichiometry of {Fe_9_P_3_}. Their species were revealed by PXRD, which showed diffraction peaks at 2*θ* = 35.6°, 43.3°, 57.3°, and 62.9° that matched well with those of γ-Fe_2_O_3_ (JCPDS card no. 39-1346). In addition, 2*θ* = 29.4° and 32.2° were assigned to the (112), (132) planes of Fe(PO_3_)_3_ (JCPDS card no. 38-0109), respectively (Fig. S5a†). High-resolution TEM images further confirmed the species of γ-Fe_2_O_3_ and Fe(PO_3_)_3_ (lattice fringe, *d* = 0.250 and 0.278 nm, respectively), which were well embedded in the OMC matrix with a particle size of around 5 nm (Fig. S5b†). In addition, we also explored the effects of dosage and pyrolysis temperature on the synchronous generation of dual-Fe/OMC, which showed the decrease of the BET surface area, pore volume, as well as the deconstruction of OMC with the increase of dosage or pyrolysis temperature (Table 1). Furthermore, the TEM images showed that the Fe-based nanoparticle size increased from 1 nm to 20 nm with increasing dosage or pyrolysis temperature (Fig. S6–S11†).

**Table 1:** A summary of the general structural information of the dual-Fe/OMC-*n-T*

Dual-Fe/OMC		BET (m^2^ g^−1^)	Pore volume (cm^3^ g^−1^)	Pore size (nm)	Particle size (nm)
*n* (mg)	*T* (°C)				γ-Fe_2_O_3_/Fe(PO_3_)_3_
6	600	711	0.36	3.4	1–2
12	600	680	0.36	3.4	1–2
18	600	671	0.34	3.4	∼3
24	600	662	0.34	3.4	∼5
30	600	550	0.27	3.4	∼20
24	700	771	0.45	3.5	∼9
24	800	849	0.48	3.5	∼15

### MRI performance of dual-Fe/OMC

**Fig. 3:**
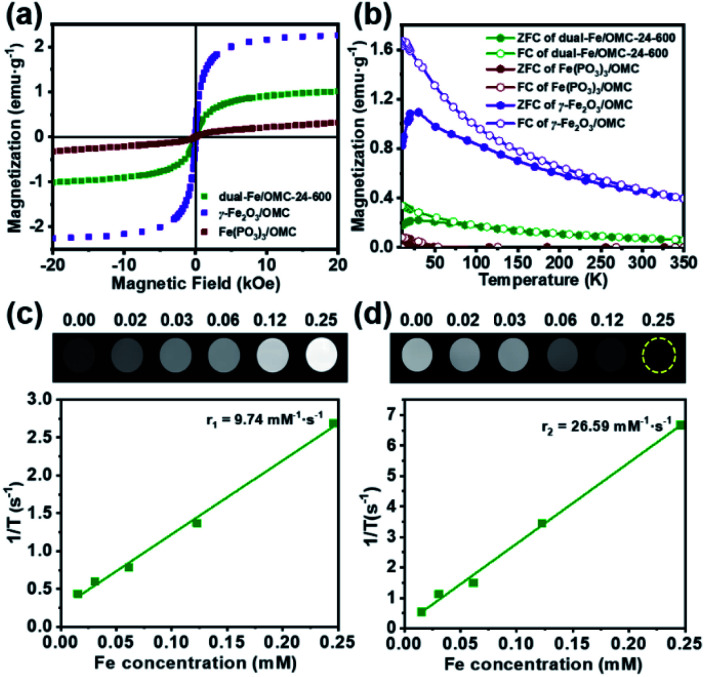
(a) The magnetic hysteresis loops and, (b) ZFC and FC magnetic curves of dual-Fe/OMC-24-600 (olive), γ-Fe_2_O_3_/OMC (purple), and Fe(PO_3_)_3_/OMC (dark red). (c) The *T*_1_-weighted MR image and (d) *T*_2_-weighted MR image of dual-Fe/OMC-24-600 in aqueous solution at 0.5 T and 32 °C.

All of the above studies demonstrate the successful preparation of dual-Fe/OMC under the concept of uniovular twins, in which the dual-Fe (γ-Fe_2_O_3_ and Fe(PO_3_)_3_) nanoparticles were *in situ* produced from {Fe_9_P_3_}, and well dispersed and embedded in the synchronously generated OMC matrix. Considering the well-dispersed dual-Fe nanoparticles available for magnetic resonance imaging (MRI) and the favourable high BET surface area for drug delivery, dual-Fe/OMC-24-600 was therefore further explored as a potential theranostic material. The magnetic properties of dual-Fe/OMC-24-600 were explored before MRI evaluation. A field-dependent magnetic study (*M*–*H* plot) at 300 K showed a S-type magnetic hysteresis loop with the magnetic remanence (*M*_r_), coercivity (*H*_c_), saturation magnetization (*M*_s_) of 0.076 emu g^−1^, 13.35 Oe and 1.07 emu g^−1^ (Fig. 3a). The thin hysteresis loop with almost zero *M*_r_ and extremely low *H*_c_ suggested a superparamagnetic behavior. The temperature-dependent magnetization measurements were also carried out in the range of 10 to 350 K at 100 Oe under zero-field-cooled (ZFC) and field-cooled (FC) conditions, which showed that the two curves were almost coincident above *T*_B_ = 30 K (Fig. 3b). To further fundamentally understand the magnetic role of the dual-Fe (γ-Fe_2_O_3_ and Fe(PO_3_)_3_) nanoparticles, γ-Fe_2_O_3_/OMC^39^ and Fe(PO_3_)_3_/OMC were prepared (Fig. S12†) and studied as comparisons, respectively. The *M*–*H* plot and ZFC/FC curves of γ-Fe_2_O_3_/OMC demonstrate a typical superparamagnetic behavior with a *M*_s_ value of 2.28 emu g^−1^. On the other hand, the *M*–*H* plot of Fe(PO_3_)_3_/OMC displayed a trend of linear increase of the magnetization with increasing magnetic field, suggesting a predominantly paramagnetic behavior (Fig. 3a and b).^40–42^ In this context, the magnetic property of dual-Fe/OMC-24-600 could be viewed as an apparent behavior of the paramagnetic Fe(PO_3_)_3_ and superparamagnetic γ-Fe_2_O_3_.

**Fig. 4:**
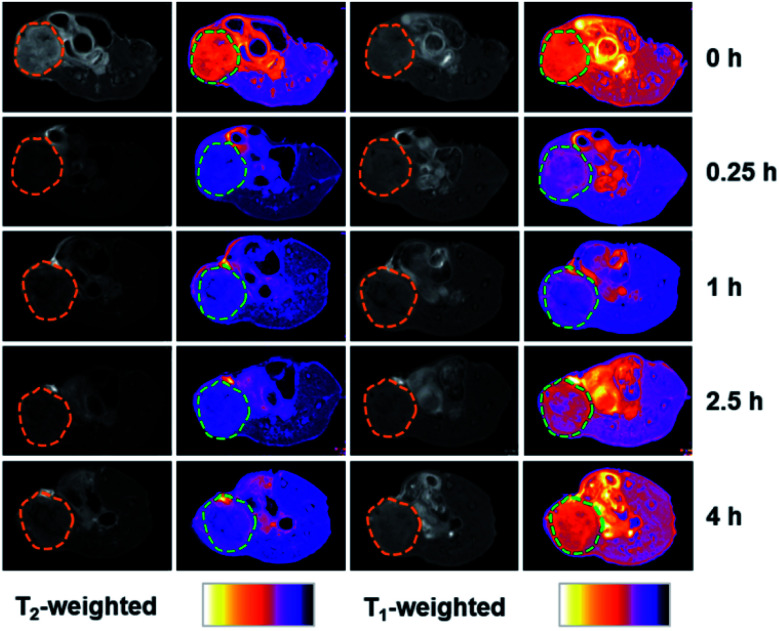
MR images of female BALB/c nude mouse bearing HeLa tumor before (0 h) and after intratumor injection of dual-Fe/OMC-24-600 after 0.25, 1, 2.5, 4 h at 7 T room temperature (tumor site at the right back of hind leg, circled as dashed line).

To further evaluate the MRI contrast effect of dual-Fe/OMC-24-600, the longitudinal (*T*_1_) and transverse (*T*_2_) relaxation times were *in vitro* measured in an aqueous solution of different Fe(iii) ion concentrations at room temperature. At *B*_0_ = 0.5 T, the *r*_1_ maps show a clear Fe(iii)-concentration dependent change from dark to bright, which is due to the shortening of the spin–lattice relaxation time with increasing Fe(iii) concentration (Fig. 3c and d). From the slope of the 1/*T*_1_ (*R*_1_) plot *versus* Fe(iii) ion concentration, *r*_1_ is estimated to be 9.74 mM^−1^ s^−1^, suggesting the effect of dual-Fe/OMC-24-600 asthe *T*_1_-weighted MRI contrast agent. The *r*_1_ values are higher than those of other MRI contrast agents (FeP, Fe@MSNs, MnFe-LDH) and commercially available Gd contrast agents, such as Gd-DTPA and Gd-DOTA.^43–47^ On the other hand, the *r*_2_ images showed a clear Fe(iii)-concentration dependent change from bright to dark, and the *r*_2_ value was calculated to be 26.59 mM^−1^ s^−1^, suggesting good sensitivity as a *T*_2_ MRI contrast agent. The *r*_2_/*r*_1_ ratio is estimated to be ∼2.7 in the range of 2–10,^48–50^ indicating the simultaneous combination of the *T*_1_ and *T*_2_ contrast effects. The inherent *T*_1_ and *T*_2_ weighted contrast in the dual-Fe/OMC sample may be related to the presence of the small magnetic nanoparticles γ-Fe_2_O_3_ and Fe(PO_3_)_3_. Following the rule of a less exploitable trend of *T*_1_ effect at higher field strengths,^51–53^ the efficiency of the *T*_1_ relaxivity decreases from *r*_1_ = 9.74 to 5.29 and 1.40 mM^−1^ s^−1^. Meanwhile, the *T*_2_ relaxivity increases from 26.59 to 62.41 and 89.33 mM^−1^ s^−1^ as *B*_0_ increases from 0.5 to 1.5 and 3.0 T (Fig. S13 and S14†). Taking the magnetic and MRI properties of Fe(PO_3_)_3_/OMC and γ-Fe_2_O_3_/OMC in consideration, the *T*_1_- and *T*_2_-weighted MR contrast effects of dual-Fe/OMC-24-600 could be ascribed to the synergistic integration of paramagnetic Fe(PO_3_)_3_ and superparamagnetic γ-Fe_2_O_3_ nanoparticles in the synchronously generated OMC matrix. The *in vivo* MRI performances were tentatively examined at 7 T on the mouse model with a subcutaneous HeLa tumor. After intratumoral injection with a dosage of 10 mg Fe per kg (Fig. 4), a darker *T*_2_ effect was recorded at *t* = 0.25 h with a signal intensity decrease of ∼70%, as compared with that before injection (*t* = 0 h). It was kept even at *t* = 4 h with a decrease of ∼55%. In contrast, the *T*_1_ effect remained dark, which is similar to that before injection (*t* = 0 h), rather than brighter images. This phenomenon could be ascribed to the high concentration of the *T*_1_ contrast agent (owing to the intratumoral injection) and the less exploitable *T*_1_ effect at 7 T (Table 2).

**Table 2:** A summary of the calculated *in vitro* relaxation rates of *r*_1_ and *r*_2_ for dual-Fe/OMC-24-600, γ-Fe_2_O_3_/OMC and Fe(PO_3_)_3_/OMC (*R*^2^: linear fitting)

Sample	*B*_0_ (T)	*r*_1_ (mM^−1^ s^−1^)	*R* ^2^	*r*_2_ (mM^−1^ s^−1^)	*R* ^2^	*r*_2_/*r*_1_
Dual-Fe/OMC-24-600	0.5	9.74	0.99	26.59	0.99	2.73
	1.5	5.29	0.98	62.41	0.10	11.80
	3.0	1.40	0.99	89.33	0.99	63.81
γ-Fe_2_O_3_/OMC	1.5	0.89	0.93	175.24	0.99	196.90
Fe(PO_3_)_3_/OMC	1.5	3.87	0.97	10.26	0.95	2.65

### *In vitro* internalization of dual-Fe/OMC inside the tumor cells

**Fig. 5:**
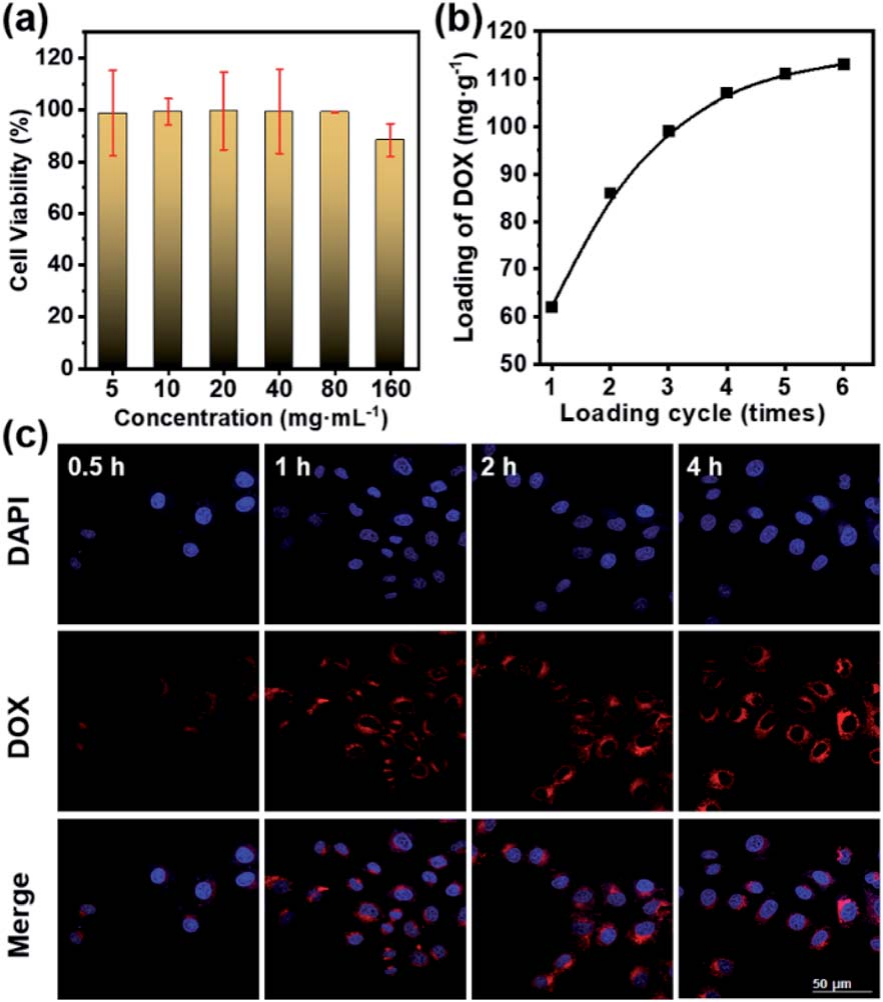
(a) Viability of HeLa cells after incubation with dual-Fe/OMC-24-600 at different concentrations. (b) Loading amount of DOX *vs.* the loading cycles in the DOX solution (1 mg mL^−1^) at room temperature. (c) CLSM images of HeLa cells incubated with DOX@dual-Fe/OMC-24-600 for 0.5, 1, 2, and 4 h. Blue and red fluorescence represent the DAPI and DOX in cells, respectively.

Before the *in vitro* internalization studies, the cytotoxicity and drug loading capacity of dual-Fe/OMC-24-600 were evaluated first. The *in vitro* cellular cytotoxicity was performed on HeLa cell lines by the cell counting kit-8 (CCK-8) assay. After 4 h incubation, the results showed that there was no significant decrease of the cell viability with increasing concentration. Even at 160 μg mL^−1^, the cell viability still remained above 88%, indicating the low toxicity of dual-Fe/OMC-24-600 within the tested concentration range (Fig. 5a). Meanwhile, the therapeutic effect of the DOX@dual-Fe/OMC-24-600 on cancer cells was also performed, which showed that apoptosis or necrosis occurred. In addition, the cell viability was decreased to 80% when the HeLa cells were incubated with higher DOX@dual-Fe/OMC-24-600 concentrations (160 μg mL^−1^) (Fig. S15†). Therefore, the reduction in the viability of HeLa cells is attributed to the DOX released from DOX@dual-Fe/OMC-24-600. Considering the OMC structure, the drug loading capacity of dual-Fe/OMC-24-600 was then studied using doxorubicin (DOX) as the probe. As recorded by the decrease of the UV-vis signals, DOX was gradually loaded into dual-Fe/OMC-24-600, resulting in the isolation of DOX@dual-Fe/OMC-24-600 with a capacity of 112 mg g^−1^ (Fig. 5b).

Based on the above results, DOX@dual-Fe/OMC-24-600 (concentration of 100 μg mL^−1^) was incubated with HeLa cells for 0.5 h, 1 h, 2 h and 4 h at 37 °C. The cellular uptake and drug release were studied by confocal laser scanning microscopy (CLSM) (Fig. 5c), which showed the blue fluorescence of DAPI staining the nuclei after 0.5 h incubation. Meanwhile, the weak red fluorescence assigned to DOX was observed in the cytoplasm, indicating the endocytosis of DOX@dual-Fe/OMC-24-600 and the initial release of DOX into the cells. With the increase of the incubation time from 0.5 h to 4 h, more stained cells were identified simultaneously with the obviously enhanced fluorescence of DOX, demonstrating a continuous DOX delivery from DOX@dual-Fe/OMC-24-600.

## Conclusions

Taking the unique advantage of the concept of uniovular twins, the {Fe_9_P_3_} cluster was synthesized and studied as the ovulum to co-assemble with resol *via* EISA method. It was then followed by the one-pot pyrolysis reaction to *in situ* produce the dual-Fe (Fe(PO_3_)_3_ and γ-Fe_2_O_3_) nanoparticles functionalized ordered mesoporous carbon (dual-Fe/OMC). Despite the less exploitable *T*_1_ effect at higher field strengths, dual-Fe/OMC was characterized by the combined *T*_1_–*T*_2_ MRI contrast effect at 0.5 T and considerable drug delivery property, owing to the synergetic integration of well dispersed paramagnetic Fe(PO_3_)_3_ and superparamagnetic γ-Fe_2_O_3_ nanoparticles in the OMC matrix. Our results demonstrate the feasibility of the concept of uniovular twins in the preparation of functional integrated OMCs, as well as shed some light on developing novel nanomaterials with combined *T*_1_–*T*_2_ dual modal MRI and DDSs for potential theranostics.

## Ethical approval

All animal procedures were performed in accordance with the Guidelines for Care and Use of Laboratory Animals of Fudan University, and approved by the Animal Ethics Committee of Fudan University.

## Conflicts of interest

There are no conflicts to declare.

## Supplementary Material

NA-002-D0NA00714E-s001
