# Supplementary material for: In situ embedding dual-Fe nanoparticles in synchronously generated carbon for the synergistic integration of magnetic resonance imaging and drug delivery
Source: Nanoscale Adv. 2020 Sep 26;2(11):5296–304. doi: 10.1039/d0na00714e (PMC9417305; doi:10.1039/d0na00714e)

Electronic Supplementary Material (ESI) for Nanoscale Advances.
This journal is © The Royal Society of Chemistry 2020

Supplementary Information for

In-situ embedding of dual-Fe nanoparticles in the synchronously generated carbon for synergistic integration of magnetic resonance imaging and drug delivery

Hui Zhang,[a] Jianping Zhang,[b] Qianqian Zhang,[a] Xiaofeng Liu,[a] Yongtai Yang,[a] Yun Ling,[a, c] and Yaming Zhou*[a]

a Shanghai Key Laboratory of Molecular Catalysis and Innovative Materials, Department of Chemistry, Fudan University, Shanghai, 200433, China.

b Department of Nuclear Medicine, Fudan University Shanghai Cancer Center, Shanghai, 200032, China.

c Zhuhai Fudan Innovation Institute, Zhuhai, Guangdong, 519000, China.

*E-mail: ymzhou@fudan.edu.cn (Prof. Dr. Zhou Y. M.)

Table S1. Structural and Textural Properties of mesoporous dual-Fe/OMC-*n*-*T* nanocomposites.

Sample	Unit Cell size (nm)	BET Surface area ($m^2 \cdot g^{-1}$)	Micropore Surface area ($m^2 \cdot g^{-1}$)	Pore Volume ($cm^3 \cdot g^{-1}$)	Micropore volume ($cm^3 \cdot g^{-1}$)	Pore Size (nm)	Wall Thickness (nm)	Fe (wt %)	P (wt %)
FDU-15	10.3	730	484	0.37	0.20	3.4	6.9	N.A.	N.A.
dual-Fe/OMC-6-600	10.7	711	475	0.36	0.19	3.4	7.3	3.05	0.56
dual-Fe/OMC-12-600	11.0	680	462	0.36	0.18	3.4	7.6	4.10	0.76
dual-Fe/OMC-18-600	11.2	671	429	0.34	0.17	3.4	7.8	6.17	1.16
dual-Fe/OMC-24-600	11.3	662	403	0.34	0.15	3.4	7.9	9.00	1.68
dual-Fe/OMC-30-600	N.A.	550	385	0.27	0.15	3.4	N.A.	11.00	2.07
dual-Fe/OMC-24-700	10.1	771	493	0.45	0.20	3.5	6.6	N.A.	N.A.
dual-Fe/OMC-24-800	10.0	849	609	0.48	0.24	3.5	6.5	N.A.	N.A.

Fig. S1 (a) Crystal structure, (b) FTIR spectra of $\{Fe_9P_3\}$, $Fe(piv)_3$ and phenylphosphonic acid ($C_6H_5PO_3H_2$), (c) TGA data of $\{Fe_9P_3\}$ clusters under N_2 and airflow from 40 to 600 ˚C, and (d) PXRD pattern of residue compounds of $\{Fe_9P_3\}$ clusters calcinated under nitrogen atmosphere.

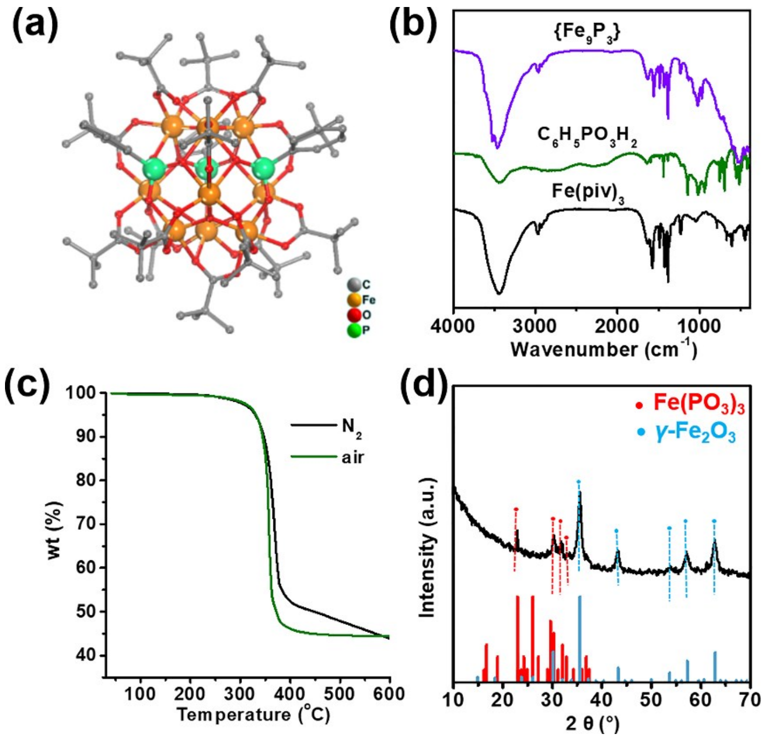

Fig. S2 (a) TEM image of dual-Fe/OMC-24-600, (b) The corresponding element analysis result of dual-Fe/OMC-24-600.

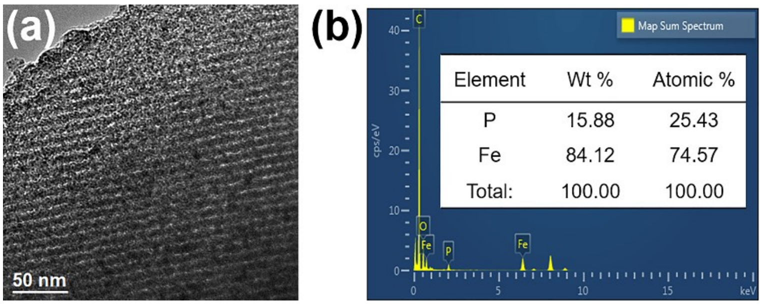

Fig. S3 The Raman spectrum of the dual-Fe/OMC-24-600. The C-C sp^3 peak of at around ~1342 cm^{-1} (D band) and the sp^2 peak at ~1590 cm^{-1} (G band).

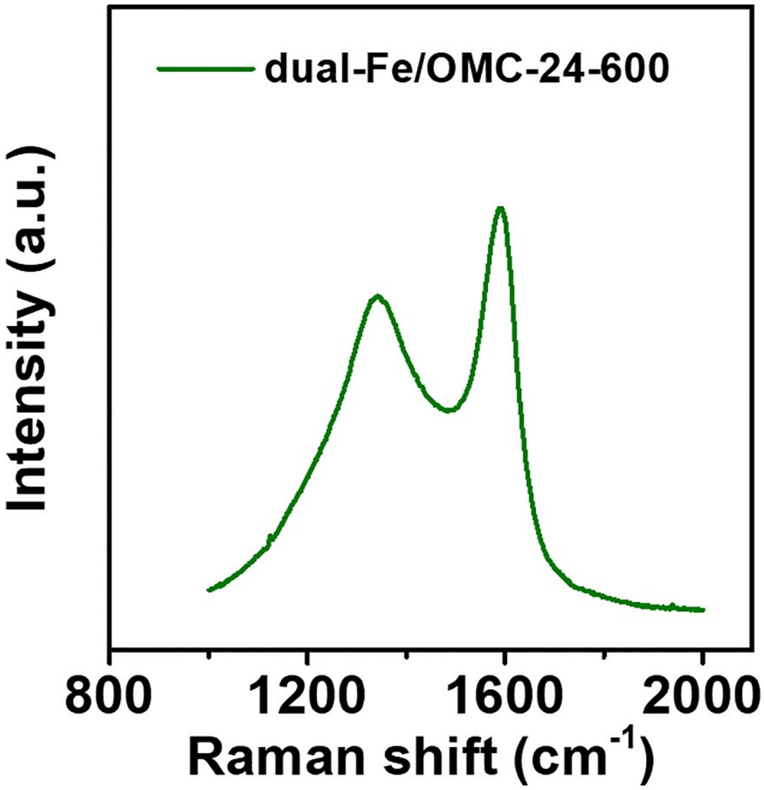

Fig. S4 (a) Fe 2p and (b) P 2p XPS spectra of the dual-Fe/OMC-24-600.

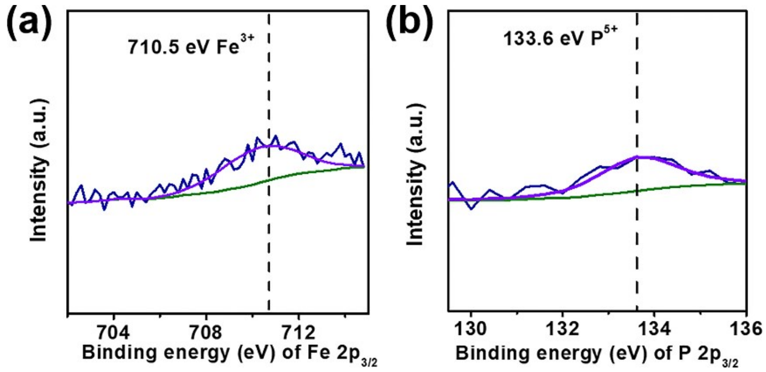

Fig. S5 (a) XRD pattern of the dual-Fe/OMC-24-600, and (b) HRTEM images. Typical lattice fringes are labeled, revealing the characteristic lattice plane distance of γ-Fe_2O_3 and $Fe(PO_3)_3$.

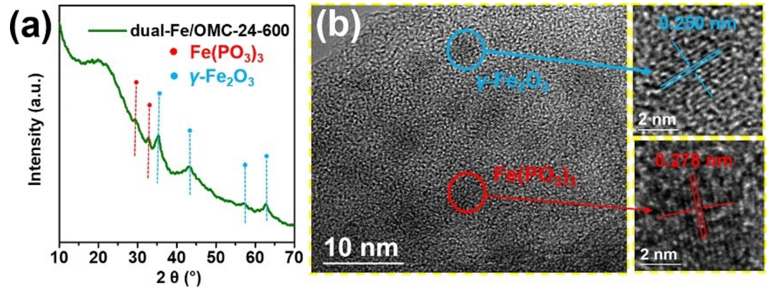

Fig. S6 (a, b) SAXS patterns for as-made mesostructured polymers and the corresponding dual-Fe/OMC-*n*-600, *n*: dose amount of {Fe_9P_3} (6 mg, 12 mg, 18 mg, 24 mg, and 30 mg), (c) N_2 adsorption−desorption isotherms and (d) the corresponding pore size distributions of dual-Fe/OMC-*n*-600 with different metal loading (6 mg, 12 mg, 18 mg, 24 mg, and 30 mg).

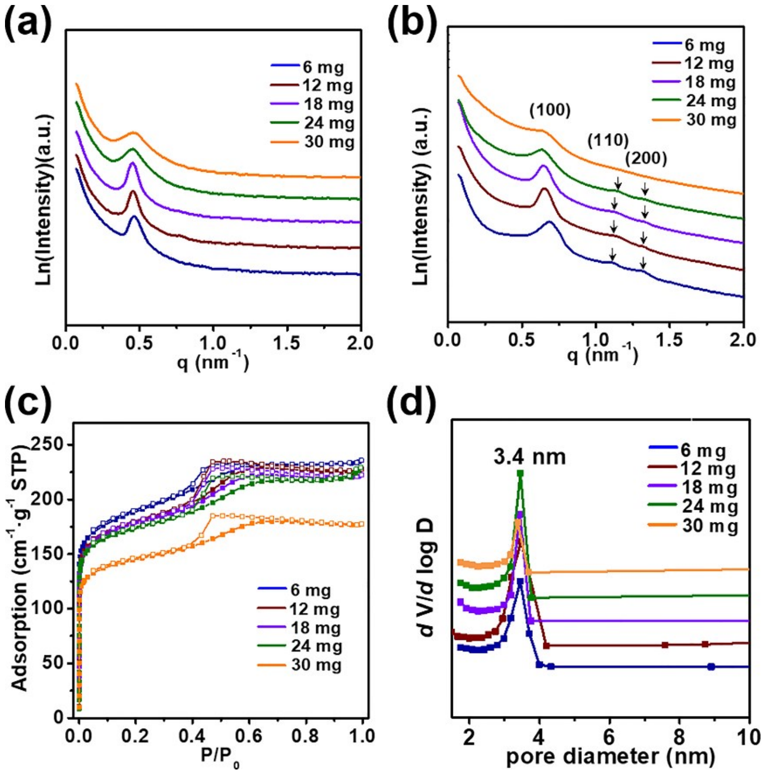

Fig. S7 TEM images of dual-Fe/OMC-*n*-600: (a) dual-Fe/OMC-6-600, (b) dual-Fe/OMC-12-600, (c) dual-Fe/OMC-18-600, (d) dual-Fe/OMC-30-600.

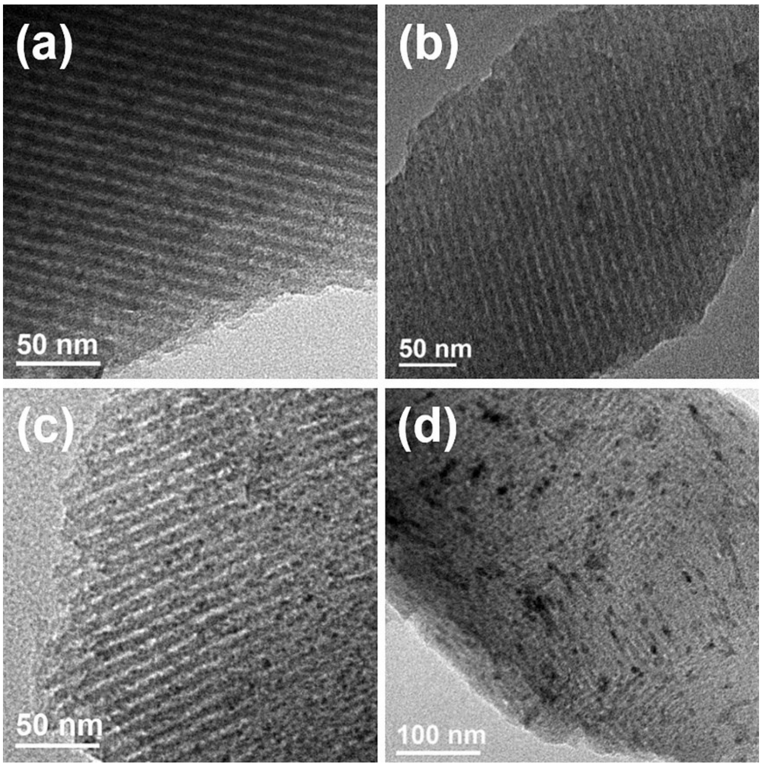

Fig. S8 PXRD patterns of dual-Fe/OMC-*n*-600. (a) dual-Fe/OMC-6-600, (b) dual-Fe/OMC-12-600, (c) dual-Fe/OMC-18-600, (d) dual-Fe/OMC-24-600, (e) dual-Fe/OMC-30-600.

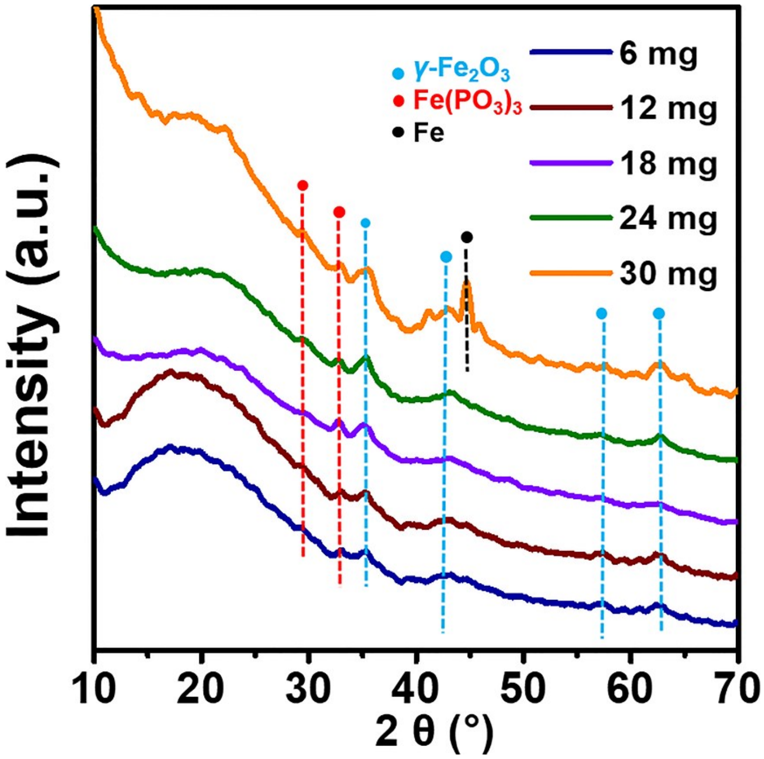

Fig. S9 Dual-Fe/OMC-24-700: (a) TEM image, (b) SAXS pattern, (c) PXRD pattern, (d) N_2 adsorption−desorption isotherm, (e) the corresponding pore size distribution, and (f) Raman spectrum.

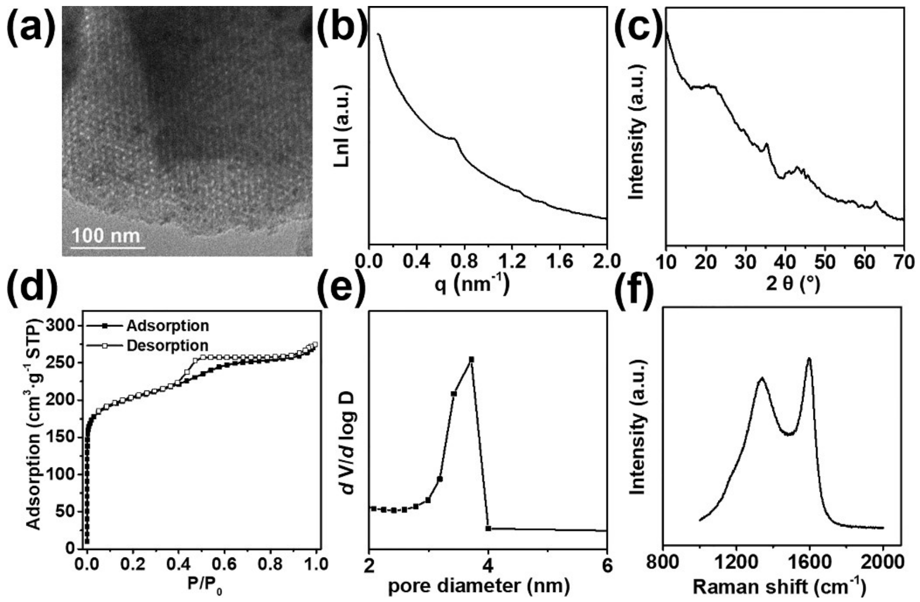

Fig. S10 Dual-Fe/OMC-24-800: (a) TEM image, (b) SAXS pattern, (c) PXRD pattern, (d) N_2 adsorption−desorption isotherm, (e) the corresponding pore size distribution, and (f) Raman spectrum.

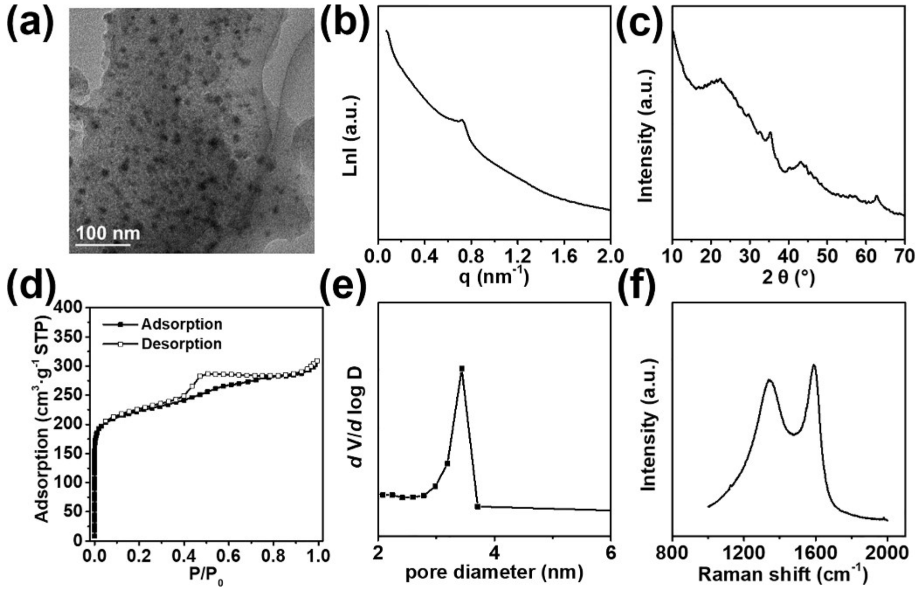

Fig. S11 (a, b) Fe-based nanoparticles particle size of dual-Fe/OMC-*n*-*T* with different metal loading: dose amount of {Fe_9P_3}: n = 0, 6, 12, 18, 24 30 mg, and T = 600, 700 and 800 °C, respectively.

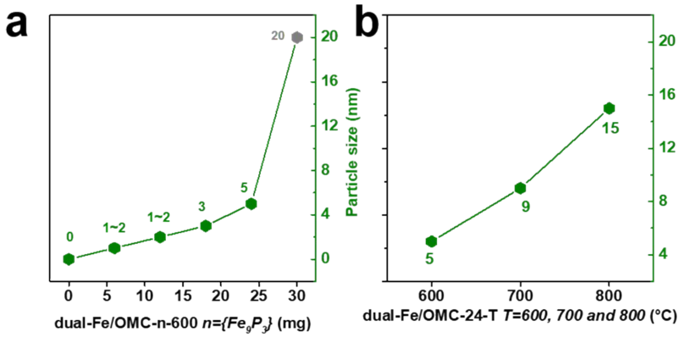

Fig. S12 (a, b) TEM images of γ-Fe_2O_3/OMC, and $Fe(PO_3)_3$/OMC, (c, d) XRD patterns of γ-Fe_2O_3/OMC, and $Fe(PO_3)_3$/OMC.

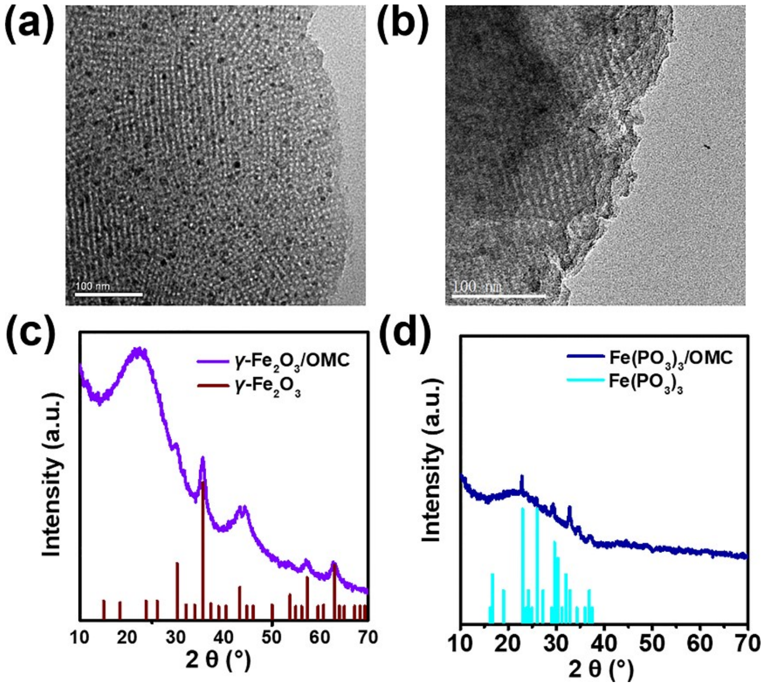

Fig. S13 (a) T_1-weighted MR image and T_2-weighted MR image, and (b) T_1, T_2 relaxivity plot of γ-Fe_2O_3/OMC in aqueous solution at 1.5 T MR system, (c) T_1-weighted MR image and T_2-weighted MR image, and (d) T_1, T_2 relaxivity plot of $Fe(PO_3)_3$/OMC in aqueous solution at 1.5 T MR system.

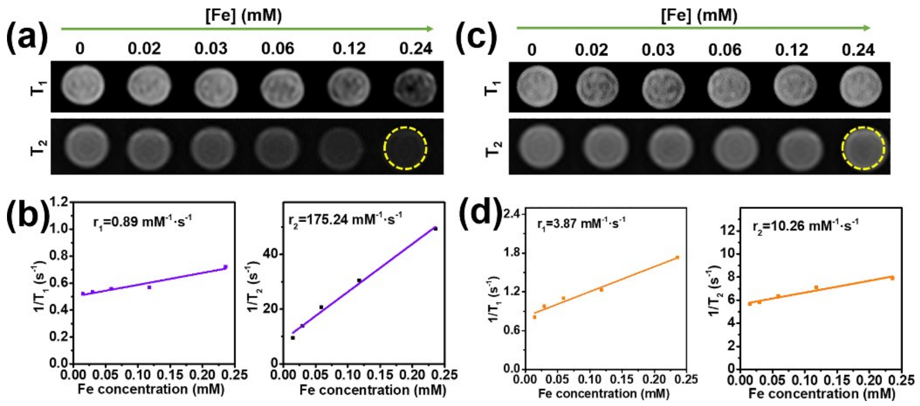

Fig. S14 (a) T_1-weighted MR image and T_2-weighted MR image, and (b) T_1, T_2 relaxivity plot of dual-Fe/OMC-24-600 in aqueous solution at 1.5 T MR system, (c) T_1-weighted MR image and T_2-weighted MR image, and (d) T_1, T_2 relaxivity plot of dual-Fe/OMC-24-600 in aqueous solution at 3.0 T MR system.

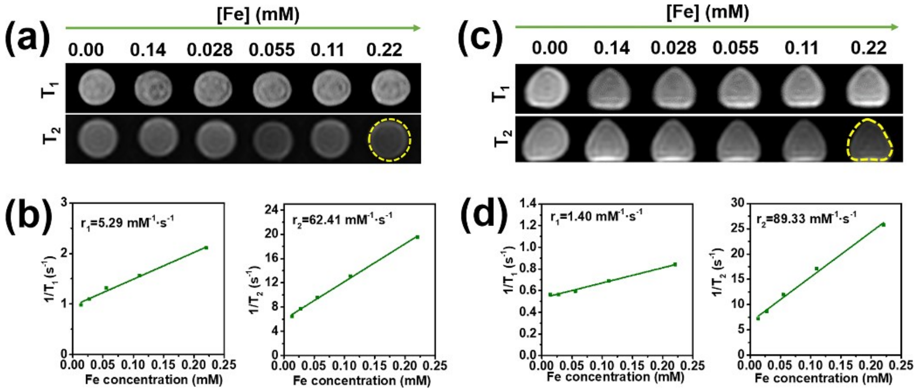

Fig. S15 The Cellular viability of after incubated for 4 h with DOX@dual-Fe/OMC-24-600 at different concentrations.

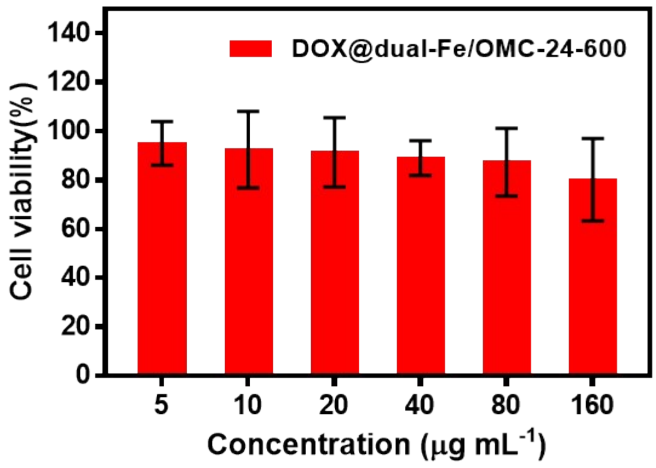

Supplement: NA-002-D0NA00714E-s001 [file NA-002-D0NA00714E-s001.pdf]
